# Direct anterior approach with conventional instruments versus robotic posterolateral approach in elective total hip replacement for primary osteoarthritis: a case–control study

**DOI:** 10.1186/s10195-024-00753-7

**Published:** 2024-02-21

**Authors:** Mattia Alessio-Mazzola, Pietro Colombo, Niccolo’ Barducci, Elena Ghezzi, Luigi Zagra, Patrizio Caldora, Marco Ometti, Giacomo Placella, Vincenzo Salini

**Affiliations:** 1 Unità di Ortopedia e Traumatologia, IRCCS Ospedale San Raffaele, Via Olgettina 60, 20132 Milan, Italy; 2 Università Vita-Salute San Raffaele, Via Olgettina 58, 20132 Milan, Italy; 3 Hip Department, IRCCS Istituto Ortopedico Galeazzi, Milan, Italy

**Keywords:** Anterior approach, Robotic arthroplasty, MAKO, Osteoarthritis, Posterolateral total hip replacement

## Abstract

**Background:**

The purpose of this study is to compare peri-operative and short-term outcomes in patients who underwent elective total hip replacement (THA) for primary osteoarthritis (OA) with direct anterior approach (DAA) versus a pair-matched cohort of patients who underwent robotic-assisted THA with posterolateral approach.

**Materials and methods:**

Data from consecutive patients who underwent elective hip replacement from 2021 to 2023 for primary OA were retrospectively retrieved and divided into two groups: the DAA group, who underwent THA with the DAA approach using conventional instruments, and the robotic posterolateral (R-PL group), who underwent robot arm-assisted THA with the posterolateral approach. Comparative assessed outcomes were: operative time, radiographical implant positioning, intake of rescue analgesics, blood loss, transfusion rate, leg length discrepancy and functional outcomes (Harris hip score and forgotten joint score).

**Results:**

A total of 100 pair-matched patients were retrieved with a mean age of 66.7 ± 10.7 (range: 32–85) years and a mean follow-up of 12.8 ± 3.6 (range: 7–24) months. No differences in patients’ characteristics were detected. Patients in the R-PL group required less rescue tramadol (*p* > 0.001), ketorolac (*p* = 0.028) and acetaminophen (*p* < 0.001). There was no significant difference in the operative time between (MD = 5.0 min; *p* = 0.071). Patients in the DAA group had significantly lower Hb levels at day 1 (*p* = 0.002) without significant differences in transfusion rate (*p* = 0.283). Patients in the R-PL group had shorter length of stay (LOS) with a mean difference of 1.8 days [*p* < 0.001; 95% confidence interval (CI) 1.4–2.3]. No difference in clinical outcomes was found [leg length discrepancy (LLD), *p* = 0.572; HHS, *p* = 0.558; forgotten joint score (FJS), *p* = 0.629]. No radiographical differences were measured in cup inclination (MD = 2.0°, *p* = 0.069), malpositioning [odd ratio (OR) = 0.2; *p* = 0.141], stem alignment (OR = 0.3; *p* = 0.485) and stem sizing (OR = 1.5; *p* = 1.000). There was no difference in complication rate except for lateral femoral cutaneous nerve damage, which was higher in DAA group (*p* < 0.001).

**Conclusions:**

R-PL and DAA THA had comparable short-term clinical and radiological outcomes along with similar complication rates. The R-PL group showed significantly lower Hb drop, rescue analgesic consumption and shorter LOS. This is a preliminary study and no strong recommendation can be provided. Further prospective randomized trials are requested to further investigate the cost-effectiveness of robotic surgery in THA.

***Level of evidence*:**

Level IV, case–control study.

## Introduction

Elective total hip replacement (THA) is the gold-standard treatment for end-stage hip osteoarthritis (OA) with satisfactory long-term results, survivorship and high patient subjective satisfaction [1, 2].

Several prosthetic designs differing in shapes, sizes, materials and tribology are currently available, but there is no clear superiority of a single specific implant [3]. However, in the last decade, there has been a notable surge in interest regarding surgical approaches, particularly increased attention in the direct anterior approach (DAA) [4, 5] and robotic surgery [6–8] to improve hip kinematics, implant positioning and clinical outcomes.

The DAA gained popularity for minimal blood loss owing to the preservation of the abductor muscles exposing the hip joint through an intermuscular plane and offering potential short-term benefits in terms of limp and pain relief [5, 9]. Furthermore, robotic-assisted THA demonstrated improved component placement and better short to mid-term outcomes compared with the conventional technique [8].

Despite the increasing amount of literature focused on both DAA and robotic-assisted THA, there is a lack of studies directly comparing clinical and radiological results of THA performed through DAA with conventional instruments and a robotic-assisted THA through the posterolateral approach.

The purpose of this study is to compare perioperative and short-term outcomes in patients who underwent elective THA for primary OA with DAA to a pair-matched cohort of patients who underwent robotic-assisted THA with a standard posterolateral approach.

## Materials and methods

This retrospective study was conducted at a single university hospital following the Strengthening the Reporting of Observational Studies in Epidemiology (STROBE) guidelines and checklist [10]. Data from consecutive patients who underwent elective hip replacement from January 2021 to January 2023 for primary OA were retrieved for internal hospital database analysis. Inclusion criteria were: diagnosis of primary hip OA with an indication of unilateral hip replacement. Exclusion criteria were: surgery performed with an anterolateral, direct lateral or posterolateral approach with conventional instruments, BMI > 25, revision cases, fractures, rheumatic disease, diabetes, previous hip surgery, bilateral cases and follow-up < 6 months. These confounders that may potentially influence operative time and outcomes were excluded to minimize bias.

Patients were divided into two groups according to approach and technique: the first group (DAA group) underwent THA with the DAA approach in a supine position using conventional instruments and was considered the control group. The second group [robot posterolateral (R-PL) group] underwent robot arm-assisted THA (Stryker—Mako total hip arthroplasty © robot) with the posterolateral approach in lateral decubitus and was considered the study group. The decision-making (DAA or R-PL) was based on surgeon preference and clinical practice. The 50 consecutive cases for each group screened for selection criteria were pair-matched. Three experienced surgeons performed all the procedures (> 100 implants per year) and data of outcomes were gathered by blinded observers not involved in surgical procedures.

Surgery was performed under spinal anaesthesia (levobupivacaine 0.5%, 10–20 mg) for all cases.

Postoperative and rehabilitation protocols were equivalent for all patients and rehabilitation started from post-operative day 1. Precisely, hip abductor strength exercises and ambulation were promoted starting on the first post-operative day and then formally prescribed daily for 60 days after surgery.

The therapeutic protocol during hospitalization was the same for all participants and included acetaminophen 1 g, per day (1 every 12 h) for 7 days and ketorolac 10 mg one per day for 3 days and enoxaparin 4000 IU every 24 h for 30 days.

Rescue painkillers were administered orally in case of residual severe pain measured as visual analogue scale (VAS) > 6. Rescue painkillers were tramadol 100 mg and ketorolac 10 mg. Moderate pain (VAS score > 3) was managed with acetaminophen 1 g.

### Surgical technique

The DAA was performed in a supine position with a standard operative table through an intermuscular and inter-nervous plane between the tensor fascia lata, sartorius and rectus femoris. Capsulectomy was performed and in-situ osteotomy of the femoral neck was completed 1 cm from the lesser trochanter. The acetabulum was exposed, reamed and a cementless trabecular titanium cup was positioned (Lima Corporate, Delta TT). Following cup positioning, the proximal femur was exposed and broached after trochanteric and calcar capsular release. Intraoperative fluoroscopy was routinely used to assess the broach stem size and alignment before the final implantation of the definitive cementless short stem (Lima Corporate, Minima S).

The PL approach was performed in lateral decubitus, and a curved incision was performed in line with the posterior aspect of the greater trochanter. The interval between the gluteus medius and the short hip rotator muscles was identified. The conjoint tendon was released and retracted to protect the sciatic nerve and quadratus femoris was preserved. The robotic-assisted procedure was completed following the standard technique of robotic arm-assisted THA (Mako Robotic-Arm assisted total hip^™^, Stryker, Warsaw, Indiana USA) and Accolade II and Trident cup (Stryker, Warsaw, Indiana USA) was implanted following surgical technique.

### Outcomes

Demographic records, operative time and X-rays were retrieved and collected in the setting of the present studies. Perioperative data were extracted from medical records, including the intake of rescue analgesics (acetaminophen, NSAIDs and opioids), blood loss, haemoglobin (Hb) drop and transfusion rate. Data were compared to assess differences between groups.

Patients were recalled to be clinically and radiographically evaluated at the final follow-up assessment by a blinded resident of orthopaedic surgery (PC) and data were collected for analysis.

Stem size and alignment were assessed by the axial alignment of the proximal femur on the AP pelvis X-rays.

Malposition was defined with a difference in the main axis of the stem > 5 mm from the anatomic femoral axis. The acetabular was evaluated by criteria described by Callanan and Lewinnek [11, 12] and cup malposition was recorded.

The leg length discrepancy (LLD) and functional outcomes were measured at the final follow-up clinical evaluation with Harris hip score (HHS) [13] and forgotten joint score (FJS) [14] and data were compared to identify differences between groups. Finally, specific complications (wound healing and neurovascular status) were investigated and clinically assessed.

### Statistical analysis

Statistical analysis was performed using IBM SPSS Statistics version 26.0 (IBM Corp., Armonk, NY, USA).

A post-hoc power calculation for two independent sample studies and continuous endpoint was performed considering the need for rescue opioids as the primary outcome measure.

The resulting power of the present study was 99.8% with a probability of type I error of 0.05 demonstrating an adequate power for the analysed sample size.

The Shapiro–Wilk test was used to identify normally distributed parameters.

Categorical variables were expressed as the absolute number of cases and percentage.

Differences between means were calculated with the *t*-test for continuous variables or with the Mann–Whitney U test if not normally distributed data. The non-parametric Wilcoxon signed rank test was used to compare continuous matched pre-operative and final data. Categorical variables were calculated using the Chi-square test or Fisher’s exact test.

Analysis of variance (ANOVA) was used to compare means of continuous normally distributed variables in two or more independent comparison groups. The Kruskal–Wallis test was used for non-normally distributed variables.

Variables achieving the *p*-value < 0.1 in univariate analysis were examined using multivariate logistic regression analysis and backward selection process. The significance threshold for tests was set at *p* < 0.05.

## Results

**Fig. 1:**
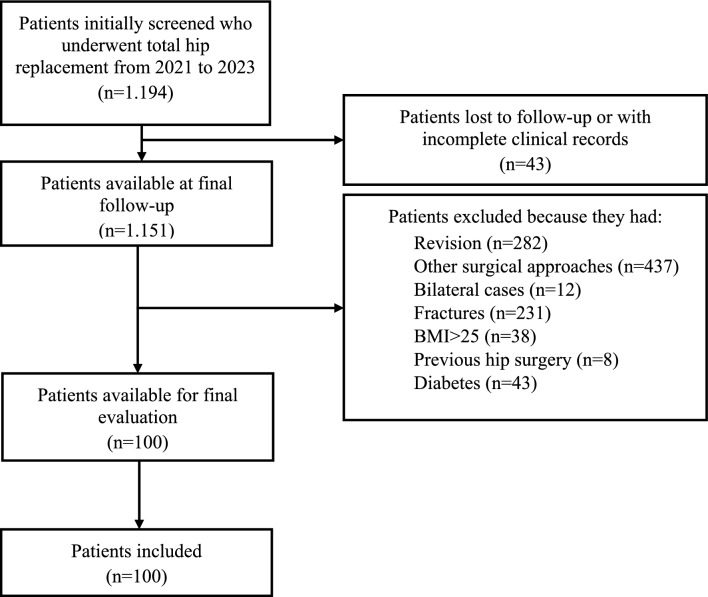
Flowchart of patient selection process

A total of 100 patients were retrieved and selected for the present study with a mean age of 66.7 ± 10.7 (range: 32–85) years and a mean follow-up of 12.8 ± 3.6 (range: 7–24) months. The flow diagram of the selection process is reported in Fig. 1 and the baseline features of the population are summarized in Table 1. No differences in patients’ characteristics were detected and both populations were comparable.

**Table 1:** Baseline features of the population included in the study

	DAA group	R-PL group	*p*-Value
Patients	50	50	–
Side (right/left)	30 (60.0%)/20 (40.0%)	26 (52%)/24 (48%)	0.216
Sex (male/female)	27 (54.0%)/23 (46.0%)	30 (60%)/20 (40%)	0.839
Age at surgery (years)	68.3 ± 10.0 (range: 42–85)	65.2 ± 11.3 (range: 32–82)	0.163
Follow-up	14.0 ± 4.7 (range: 7–24)	11.6 ± 1.2 (range: 9–16)	0.060
Pre-operative Hb level	13.9 ± 1.8 (range: 8.1–18.0)	14.5 ± 1.6 (range: 10.1–18.3)	0.079

Perioperative clinical outcomes are reported in Table 2.

**Table 2:** Details of perioperative outcomes during hospitalization

	DAA group	R-PL group	*p*-Value	
Rescue tramadol (mg)	480 ± 321 (range: 0–1300)	193 ± 230 (range: 0–700)	< 0.001	*
Rescue acetaminophen (g)	8.7 ± 2.9 (range: 0–12)	6.2 ± 2.3 (range: 0 to 10)	< 0.001	*
Rescue ketorolac (mg)	188.4 ± 134.4 (range: 0–420)	139.2 ± 79.2 (range: 0–300)	0.028	*
Operative time (min)	67.3 ± 15.7 (range: 45–134)	62.1 ± 12.4 (range: 40–95)	0.071	
Hb day 1	11.3 ± 1.6 (range: 8.1–15.2)	12.5 ± 1.8 (range: 6.7–15.9)	0.002	*
Hb drop	2.5 ± 1.1 (range: − 0.9 to 5.6)	2.0 ± 0.8 (range: 0.2–3.8)	0.012	*
Number of transfusions	0.1 ± 0.5 (range: 0–2)	0.1 ± 0.3 (range: 0–2)	0.283	
Length of stay	4.3 ± 1.4 (range: 3–8)	2.5 ± 0.6 (range: 2–5)	< 0.001	*

Asterisks highlights significant differences. Continuous variables are expressed with main values, standard deviation and range of values (under parenthesis)

Absolute values are expressed by frequencies and relative percentages (under parenthesis)

Analysis of clinical records revealed that patients in the R-PL group required less rescue tramadol (*p* > 0.001), ketorolac (*p* = 0.028) and acetaminophen (*p* < 0.001) during hospitalization compared with those in the DAA group. The analysis of confounders detected that patient sex and age at surgery did not influence analgesic consumption (*p* > 0.05).

There was no significant difference in the operative time between the two groups (MD = 5.0 min; *p* = 0.071).

Patients in the DAA group had significantly lower Hb levels at day 1 (*p* = 0.002) and higher Hb drop (*p* = 0.012) without significant differences in transfusion rate during hospitalization (*p* = 0.283). Furthermore, patients in the R-PL group had shorter LOS with a mean difference of 1.8 days [*p* < 0.001; 95% confidence interval (CI) 1.4 to 2.3].

The final follow-up assessment revealed no difference in clinical outcome between the two groups (HHS, *p* = 0.558; FJS, *p* = 0.629).

A total of 100 anteroposterior pelvic X-rays were assessed to evaluate implant position and LLD with digital measurement. No differences were detected in cup inclination (MD = 2.0°, *p* = 0.069), cup malpositioning [odds ratio (OR) = 0.2; *p* = 0.141], stem alignment (OR = 0.3; *p* = 0.485), stem sizing (OR = 1.5; *p* = 1.000) and LLD (MD = 0.2 mm; *p* = 0.572). Functional scores and radiographic assessment performed at the final follow-up assessment are summarized in Table 3.

**Table 3:** Details of final follow-up assessment including clinical, functional scores and radiographic outcomes

	DAA group	R-PL group	*p*-Value
Leg length discrepancy (mm)	0.4 ± 1.4 (range: − 2 to 3)	0.6 ± 1.4 (range: − 2 to 5)	0.572
Harris hip score (points)	79.1 ± 19.0 (range: 28–91)	81.6 ± 17.4 (range: 1–91)	0.558
Forgotten joint score (points)	4.7 ± 8.9 (range: 0–12)	5.7 ± 10.1 (range: 0–48)	0.629
Cup inclination (degrees)	45.1 ± 6.1 (range: 22–58)	43.0 ± 3.9 (range: 34–52)	0.069
Cup malalignment	2 (4.0%)	0 (0.0%)	0.141
Stem appropriate size	49 (98.0%)	48 (96.0%)	1.000
Stem malalignment	1 (2.0%)	0 (0.0%)	0.485

Continuous variables are expressed with main values, standard deviation and range of values (under parenthesis)

Absolute values are expressed by frequencies and relative percentages (under parenthesis)

Post-operative complications are reported in Table 4. There was no difference in infection rate (*p* = 0.594), wound healing (*p* = 0.596) and post-operative anaemia (< 8 mg/dL; *p* = 0.232). No patients sustained hip dislocation during the follow-up period. Lateral femoral cutaneous nerve damage was present in 38.3% of patients in the DAA group (*p* < 0.001).

**Table 4:** Details of complications that occurred during the follow-up period

	DAA group	R-PL group	Odds ratio	*p*-Value
Infection	1 (2.0%)	2 (4.0%)	1.9	0.594
Meralgia paresthetica	18 (36.0%)	0 (0.0%)	0.1	< 0.001
Delayed wound healing	3 (6.0%)	2 (4.0%)	0.6	0.596
Severe anaemia	2 (4.0%)	1 (2.0%)	0.5	0.232
Dislocation	0 (0.0%)	0 (0.0%)	1.0	1.000

Absolute values are expressed by frequencies and relative percentages (under parenthesis)

## Discussion

The main finding of the study indicates that the R-PL and DAA THA had comparable short-term clinical and radiological outcomes, along with similar complication rates. Nevertheless, statistically significant differences were identified regarding multiple investigated parameters, precisely, patients in the R-PL group notably required fewer rescue opioids, acetaminophen and NSAIDs compared with the DAA group during hospitalization, resulting in a shorter LOS.

Historically, the DAA was considered to be less painful than the posterolateral approach [15, 16] but available studies do not report comparative results of robotic arm-assisted surgery. The current research addressed potential confounders related to sample populations (i.e. age and sex), excluding the influence of patient specific features on pain level and drug metabolism, by demonstrating that the robotic technique is less painful in the post-operative period. This finding can be clarified by the minimized invasiveness of robotic arm-assisted THA, specifically concerning cup reaming.

In contrast to literature [17–19], the present study demonstrates that the R-PL approach is comparable to DAA in terms of operative time with a mean difference of 5 min. We emphasize that all the procedures were performed by experienced surgeons with proven experience in hip arthroplasty and robotic surgery. We also highlight that the operative time of R-PL can be increased only if compared with the traditional posterolateral approach performed by expert surgeons [20].

This study showed a significantly lower Hb drop in the R-PL group compared with the DAA group as well as a higher Hb value at post-operative day 1. Despite the overall reduction of blood loss, which supports the reduced surgical invasiveness of the robotic technique, the transfusion rate was the same for the DAA and R-PL groups, indicating limited blood loss also in the DAA group [7]. Furthermore, the higher Hb drop and lower Hb level of post-operative day 1 of the DAA group can be justified by the lower, although not significant, peri-operative Hb level of this group.

Enhanced recovery following THA played a key role in the last decade and the LOS has been evaluated as an important outcome measure in the latest studies on robotic-assisted arthroplasty [6, 21, 22]. A retrospective cohort study comparing MAKO (*n* = 56) with standard surgery (*n* = 51) showed that the robotic-assisted system was associated with shorter LOS [21]. The results of the present research agree with the latest evidence in literature, suggesting that specific robotic preoperative planning tailored to a patient’s anatomy can further promote pain control. Rapid patient discharge is also associated with excellent pain control, supporting the data in this study on the diminished requirement for analgesics during hospitalization in the R-PL group.

No significant mid-term differences were found regarding clinical outcomes assessed by HHS and FJS showing that both techniques achieved excellent clinical outcomes at 1 year. Accordingly, experienced surgeons can achieve the same results performing the traditional technique. The literature reports contrasting findings related to functional outcomes in robotic THA, but the meta-analysis confirmed no significant differences between manual and robotic THA, substantially confirming the results of the present study [6, 22, 23].

Component positioning in THA is essential to ensure joint stability and long-term survivorship of prosthetic implants. Implant malpositioning is associated with a higher risk of complications, including impingement, dislocation, wear and revision [24, 25]. The current study demonstrated that cup inclination and stem sizing were comparable and experienced surgeons achieved equivalent radiological results as robotic-assisted hip arthroplasty with a mean difference of 2°. Despite these findings, meta-analyses [26–28] reported an improved radiological positioning with the robotic technique, but the participation of less experienced surgeons among the operators could influence the radiological results justifying this difference.

The LLD following a THA is still a debated point and literature reported contrasting findings with different hip approaches [29–31]. The present study reported no significant differences in LLD at 1 year follow-up, and the results are confirmed by other authors [29, 30] who demonstrated comparable LLD in R-PL THA and DAA. The reasons for the accuracy of the DAA can be related both to the surgeon’s experience and to the supine position used in DAA that provides an intuitive intra-operative assessment of leg length.

Post-operative complications during the follow-up period were also assessed and compared but no significant differences were found except for meralgia paresthetica which typically affects 30% of hip replacement with anterior approach [32]. In this series, 36% of patients in the DAA group had lateral femoral cutaneous nerve palsy and results are comparable with those reported in the literature focused on this complication [32].

The infection rates were comparable between R-PL and conventional THA; however, it is important to highlight that robotic surgery exhibited a 4% infection rate (over 2% of DAA), higher than the one reported in literature [33], raising concern regarding the request of additional healthcare personnel as biomedical engineers potentially increasing the risks of contamination. In addition, the proper draping preparation and use of the robot can also represent a potential criticism related to prosthetic joint infection.

There is a lack of studies directly comparing clinical and radiological results of THA performed through DAA with conventional instruments and a robotic-assisted THA through the posterolateral approach. To the best of our knowledge, this is the first study directly comparing R-PL and DAA in a single centre with standardized protocol and expert surgeons.

Additional strengths are the precise design (retrospective, with a pair-matched cohort of patients), the limited number of surgeons involved (three expert surgeons performing more than 100 hip replacements per year), the single-institution series and data collected by blinded observers not involved in the surgical procedures. Furthermore, strict inclusion and exclusion criteria were applied to address and evaluate potential confounders.

Limitations of this study are the limited sample size, the retrospective design, and the limited follow-up period reporting only short to mid-term follow-up results. Furthermore, the two techniques require different implants, precisely short cementless stems were used for DAA and standard cementless collarless Corail-type stems for the R-PL technique, and this was related to the robotic technique. Moreover, no cost-effectiveness analysis was performed to further assess robotic surgery in total hip replacement.

## Conclusions

R-PL and DAA THA had comparable short-term clinical and radiological outcomes along with similar complication rates. The R-PL group showed significantly lower Hb drop, rescue analgesic consumption and shorter LOS. This is a preliminary study and no strong recommendation can be provided. Further prospective randomized trials are requested to further investigate the cost-effectiveness of robotic surgery in THA.

## Data Availability

Data are available only upon reasoned requested to the corresponding authors.

## Abbreviations

DAA: Direct anterior approach
LLD: Leg length discrepancy
NSAIDs: Non-steroidal anti-inflammatory drugs
OA: Osteoarthritis
LOS: Length of stay
THA: Total hip arthroplasty
R-PL: Robotic posterolateral
VAS: Visual analogue scale
